# New preparation methods for coated heat exchangers in adsorption refrigeration and heat pumps applications

**DOI:** 10.1038/s41598-022-11548-3

**Published:** 2022-05-14

**Authors:** Oscar Banos, Ute Bergmann, Maja Glorius, Sven Ohmann, Torsten Seidel, Cornelia Breitkopf

**Affiliations:** 1 Chair of Technical Thermodynamics, Faculty of Mechanical Science and Engineering, Institute of Power Engineering, Technische Universität Dresden, 01069 Dresden, Germany; 2 Chair of Biomaterials, Faculty of Mechanical Science and Engineering, Institute of Material Science, Technische Universität Dresden, 01069 Dresden, Germany; 3 Fraunhofer Institute for Manufacturing Technology and Advanced Materials, 25389 Dresden, Germany

**Keywords:** Energy science and technology, Engineering, Materials science

## Abstract

Adsorption refrigeration systems and heat pumps still possess a relatively reduced market share as compared to the traditional compression systems. Despite having the great advantage of being powered by cheap heat (instead of expensive electric work), the implementation of systems based on adsorption principles remains limited to few specific applications. The main drawback that needs to be solved is their reduced specific power due to the low thermal conductivity and low stability of the adsorbents. The current state of the art of commercial adsorption cooling systems rely on adsorbers based on coated finned heat exchangers to optimize the cooling power. It is a well known result, that the reduction of the thickness of the coating derives in a reduction of the mass transport impedance, and that the increment of the ratio surface to volume of conductive structures increases the power without reducing the efficiency. The metallic fibres used in this work can offer a ratio of specific surface in the range of 2500–50,000 m^2^/m^3^.Three methods of preparing very thin but stable salt-hydrate coatings on metallic surfaces, including metallic fibres, for the production of coated heat exchangers with high specific power, are presented for the first time. A surface treatment based on aluminium anodizing was chosen to create a stronger bond between coat and substrate. The microscopic structure of the resulting surface was analysed by Scan Electron Microscopy. To verify the presence of the desired species Attenuated Total Reflectance-Fourier Transformed Infrared and Energy dispersive X-ray spectroscopy were employed in the analysis. Their capacity to form hydrates was verified via simultaneous Thermogravimetric Analysis (TGA)/Differential Thermogravimetry (DTG). Over a mass difference of 0.07 g(water)/g(composite) was detected in the coating of MgSO_4_, which showed signs of dehydration at temperatures around 60 °C, and repeatability after rehydration. Also positive results were obtained with SrCl_2_ and ZnSO_4_ with mass differences around 0.02 g/g below 100 °C. Hydroxyethyl Cellulose was chosen as additive to increase the stability and adherence of the coatings. The adsorption properties of the product were evaluated with simultaneous TGA-DTG, while their adherence was characterized by means of a procedure based on the test described in ISO2409. Coatings of CaCl_2_ displayed a much improved consistency and adherence, while retaining its adsorption capacity, showing mass differences of around 0.1 g/g at temperatures below 100 °C. Also MgSO_4_ retains the capacity of forming hydrates, showing a mass difference of more than 0.04 g/g below 100 °C. Finally, coated metallic fibres were investigated. Results show that the effective heat conductivity of a fibre structure coated with Al_2_(SO_4_)_3_ can be up to 4.7 times higher as compared to a block of pure Al_2_(SO_4_)_3_ . The coverage of the pursued coatings was visually investigated and the internal structure was evaluated by microscopic imaging of cross-sections. Coatings of around 50 µm of Al_2_(SO_4_)_3_ were generated, but in general the process requires optimization to achieve a more uniform distribution.

## Introduction

Adsorption systems have drawn a lot of attention during the last decades because they represent an environmentally friendly alternative to traditional compression heat pumps or refrigeration systems. With comfort standards and average global temperatures increasing, adsorption systems have the potential to reduce the dependence on fossil fuels in the near future. Besides, any improvement in the fields of adsorption refrigeration or heat pumps can be transferred to the field of thermal energy storage, which constitutes an additional increment in the capacity to make an efficient use of primary energy. The main advantage of adsorption heat pumps and refrigeration systems is that they can be processed by low quality heat. This makes them suitable for low temperature sources like solar energy or waste heat. Regarding energy storage applications, adsorption possesses the advantages of its higher energy density and its lower energy dissipation for long term applications as compared to sensible or latent heat storage.

Adsorption heat pumps and refrigeration systems follow similar thermodynamic cycles as those followed by their vapor-compression counterparts^1^. The main difference is the substitution of the compressor part by an adsorber. This element, when maintained at medium temperature, is capable of adsorbing low-pressure vapor refrigerants, which enables the evaporation of higher amount of refrigerant even when the liquid is at low temperatures. It is necessary to ensure a continuous cooling of the adsorber to remove the enthalpy of adsorption (exothermic). The adsorber is regenerated at high temperature, which forces desorption of vapor refrigerant. Heat must be continuously added to supply the enthalpy of desorption (endothermic). As adsorption events are characterized by changes of the temperature, a high thermal conductivity is necessary to obtain high specific powers. However, the low heat conductivity is a major drawback so far in most of the applications.

The main challenge with respect to conductivity consists in the increment of its average value, and at the same time to ensure the maintenance of transport paths that allow the flow of the adsorbed/desorbed vapor. Two approaches have been usually adopted to achieve this goal: composites and coated heat exchangers. The most popular and successful composites are those that use carbon-based additives, i.e., expanded graphite, activated carbon, or carbon fibers. Oliveira et al.^2^ impregnated calcium chloride in expanded graphite powder to produce an adsorber with a specific cooling power (SCP) up to 306 W/kg and a Coefficient of Performance (COP) up to 0.46. Zajaczkowski et al.^3^ proposed a combination of expanded graphite, carbon fibers and calcium chloride with an overall conductivity of 15 W/mK. Jian et al.^4^ tested composites with Expanded Natural Graphite Treated with Sulfuric Acid (ENG-TSA) as substrate in two-stage adsorption refrigeration cycles. Models predicted COPs between 0.215 and 0.285 with SCPs between 161.4 and 260.74 W/kg.

Until now the approach that constitutes the most feasible solution is given by coated heat exchangers. The mechanisms to coat those heat exchangers can be divided into two categories: direct synthesis and binders. The most successful method is the direct synthesis, which consists in the formation of the adsorbent material, out of the corresponding reactants, directly on the surface of the heat exchanger. Sortech^5^ patented a synthesis method for coated zeolite that is employed in one of the lines of chillers commercialized by the company Fahrenheit GmbH. Schnabel et al.^6^ tested the performance of two zeolites coated on stainless steel. However, this method works only with specific adsorbents, which makes coating with binders an interesting alternative. Binders are passive species, selected to maintain the adherence and/or mass transport of the adsorbent, but play no role in the adsorption process or the increment of the conductivity. Freni et al.^7^ coated an aluminum heat exchanger with zeolite AQSOA-Z02 stabilized by a clay-based binder. Calabrese et al.^8^ have investigated the preparation of zeolite coatings with a polymeric binder. Ammann et al.^9^ presented a method to create a porous zeolite coating with a magnetic mixture of polyvinyl alcohol. Aluminum oxide (alumina) has been also used as binder in adsorbers^10^. To the best of our knowledge, cellulose and hydroxyethyl cellulose have been used only in combination with physical adsorbers^11,12^. Sometimes binders are not used in coatings but to create a structure by themselves^13^. An alginate-derived polymer matrix was combined with several salt hydrates to create a flexible structure of composite beads, preventing leakage during deliquescence, and allowing sufficient mass transport^14^. Clays like bentonite and attapulgite have been employed as binder for the preparation of composites^15–17^. Ethyl cellulose has been used to micro encapsulate calcium chloride^18^, or sodium sulfide^19^.

Composites with porous metallic structures could be classified halfway between additives and coated heat exchangers. The high ratios of surface-to-volume characteristic of these structures are an advantage. This results into bigger contact surfaces between adsorbent and metal without increasing the inert mass, which would decrease the overall efficiency of the refrigeration cycle. Lang et al.^20^ increased the overall conductivity of a zeolite adsorber with an aluminum honeycomb structure. Gillerminot et al.^21^ intensified the thermal conductivity of a NaX zeolite bed with copper and nickel foams. Even though the composites were used as phase change materials (PCMs) the conclusions from Li et al.^22^ and Zhao et al.^23^ are also of interest for chemical adsorption. They compared the performance of expanded graphite and metallic foams, concluding that the later are preferred only in those cases where corrosion is not an issue. Other metallic porous structures were recently compared by Palomba et al.^24^. Metallic salts embedded in metallic foams were investigated by van der Pal et al.^25^. All the previous examples correspond to compact beds of granulate adsorbent. Metallic porous structures have been scarcely employed in coated adsorbers which is a more optimized solution. An example can be found in the publication of Wittstadt et al.^26^ in combination with zeolites, but no attempts could be found in combination with salt hydrates in spite of their higher energy density^27^.

Therefore, in this paper three methods for the preparation of adsorbent coatings will be investigated: (1) a coating with binder, (2) a direct reaction, and (3) a surface treatment. Hydroxyethyl cellulose is the binder selected in this work because of the previously reported stability and good adherence of the coatings in combination with physical adsorbents. This method is initially investigated for coatings of flat surfaces, and then applied to metal fiber structures. A preliminary analysis of the possibilities offered by chemical reactions to generate adsorbent coatings has been already previously reported^28^. That previous experience is now transferred to coatings of metallic fibrous structures. The surface treatment selected for this work is a method based on aluminum anodizing. Aluminum anodizing has been already successfully used in combination with metallic salts for aesthetic purposes^29^. In those cases, very stable and resistant coatings can be generated. However, they are not able to perform any adsorption or desorption processes. In this work a variation of this method is presented, that allows the transport of mass, while profiting from the adhesion properties of the original process. To the best of our knowledge none of the methods described here have been previously investigated. They constitute novel techniques of great interest because they allow the formation of adsorbent coatings with salt hydrates, which present several advantages as compared to the frequently investigated physical adsorbents.

## Experimental

### Materials

The punched aluminum plates used as substrate for these experiments were provided by ALINVEST Břidličná, Czech Republic. They contain 98.11% Al, 1.3622% Fe, 0.3618% Mn and traces of Cu, Mg, Si, Ti, Zn, Cr, and Ni.

The materials chosen for the preparation of the composites were selected according to their thermodynamic properties, more specifically, they were selected based on the amount of water that they can adsorb/desorb at temperatures below 120 °C.

Magnesium sulfate (MgSO_4_) is one of the most interesting and investigated salt hydrates^30–41^. Its thermodynamic properties have been systematically measured proving its suitability for applications in the fields of adsorption refrigeration, heat pumps, and energy storage. Magnesium sulfate dry CAS-Nr. 7487-88-9 99% was employed (Grüssing GmbH, Filsum, Niedersachsen, Germany).

Calcium chloride (CaCl_2_) (H319) is another deeply investigated salt because of the interesting thermodynamic properties of its hydrates^41–44^. Calcium chloride hexahydrate CAS-Nr. 7774-34-7 97% was employed (Grüssing, GmbH, Filsum, Niedersachsen, Germany).

Zinc sulphate (ZnSO_4_) (H302, H318, H410) and its hydrates possess thermodynamic properties suitable for adsorption processes at low temperatures^45,46^. Zinc sulphate heptahydrate CAS-Nr. 7733-02-0 99.5% was employed (Grüssing GmbH, Filsum, Niedersachsen, Germany).

Strontium chloride (SrCl_2_) (H318) has also interesting thermodynamic properties^4,45,47^, although it has been frequently investigated in combination with ammonia for application in adsorption heat pumps or energy storage. Strontium chloride hexahydrate CAS-Nr. 10,476-85-4 99.0–102.0% was employed in the synthesis (Sigma Aldrich, Saint Louis, MO, USA).

Copper sulphate (CuSO_4_) (H302, H315, H319, H410) is not one of the hydrates frequently found in the specialized literature, even though its thermodynamic properties are of interest for application at low temperatures^48,49^. Copper sulphate CAS-Nr. 7758-99-8 99% was employed in the synthesis (Sigma Aldrich, Saint Louis, MO, USA).

Magnesium chloride (MgCl_2_) is one of the salt hydrates which has lately received more attention in the field of thermal energy storage^50,51^. Magnesium chloride hexahydrate CAS-Nr. 7791-18-6 pure, pharma grade was employed in the experiments (Applichem GmbH., Darmstadt, Germany).

As mentioned above, hydroxyethyl cellulose was chosen because of the positive results in similar applications. The material used in our synthesis is Hydroxyethyl-cellulose CAS-Nr 9004-62-0 (Sigma Aldrich, Saint Louis, MO, USA).

Metal fibers are prepared from short metallic filaments joined together by compression and sintering, in a process called crucible melt extraction (CME)^52^. This means that their thermal conductivity is not only determined by the bulk conductivity of the metal employed in the fabrication and the porosity of the final structure, but also by the quality of the bonds between filaments. The fibres are not isotropic, during the production the fibres tend to be allocated in a certain direction, which makes the thermal conductivity in the cross direction much lower.

### Analysis

A simultaneous Thermogravimetric Analysis (TGA)/Differential Thermogravimetry (DTG) vacuum-tight device (Netzsch TG 209 F1 Libra) was used to investigate the water sorption properties. The measurements were carried out in a flowing nitrogen atmosphere with a flow rate of 10 ml/min and a temperature ranged from 25 to 150 °C in an alumina crucible. The heating rate was 1 °C/min, where the sample mass varied between 10 and 20 mg with a resolution of 0.1 µg. It must be noticed in this work, that the values of the mass differences given per unit of surface are subjected to big uncertainty. The samples used in the TGA-DTG are very small and unevenly cut, which makes the determination of their area imprecise. The extrapolation of those values to bigger areas can be done only with the precaution of considering big deviations.

Attenuated Total Reflectance-Fourier Transformed Infrared (ATR-FTIR) spectra were obtained using a Platinum ATR accessory (Bruker Optik GmbH, Germany) in a Bruker Vertex 80 v FTIR spectrometer (Bruker Optik GmbH, Leipzig, Germany). Spectra of the clean, dry diamond crystal in vacuum measured directly before the samples were used as background for the experimental measurements. Samples were measured in vacuum, the spectral resolution used was 2 cm ^−1^ and the number of averaged scans was 32. The wavenumber range was from 8000 to 500 cm^−1^. The analysis of the spectra was done with OPUS software.

SEM analysis was carried out with a DSM 982, Gemini from Zeiss with an acceleration voltage of 2 and 5 kV. Energy dispersive X-ray spectroscopy (EDX) was provided with help of a Thermo Fischer System 7 with a Peltier cooled silicon-drift-detector (SSD).

### Hydroxyethyl cellulose (HEC)

The preparation of the metallic plates was made following a process similar to the one described in^53^. The plates where first immersed in sulphuric acid 50% vol. for 15 min. Then, they were introduced around 10 s in sodium hydroxide 1 M. The samples where then washed with abundant distilled water, followed by a 30 min bath in distilled water. After the surface pre-treatment the samples where immersed in a saturated solution with 3% wt. HEC and the target salt. Finally, they were taken out and dried at 60 °C.

### Anodic oxidation

The method of anodic oxidation intensifies and strengthens the oxide layer naturally generated on passivating metals. A sulfuric acid anodic oxidation of aluminum plates was carried out under intensified conditions and subsequently followed by a sealing process in hot water. The anodization followed an initial etching treatment using 1 mol/l NaOH (600 s) with subsequent neutralizing in 1 mol/l HNO_3_ (60 s). The electrolyte solution was a mixture of 2.3 M H_2_SO_4_, 0.01 M Al_2_(SO_4_)_3_ and 1 M MgSO_4_ + 7H_2_O. The anodization was carried out at (40 ± 1) °C, 30 mA/cm^2^ for 1200 s. The sealing process was performed in different saturated saltic solutions, as described under Materials (MgSO_4_, CaCl_2_, ZnSO_4_, SrCl_2_, CuSO_4_, MgCl_2_). The specimens were boiled therein for 1800s.

## Results and discussion

Three different methods for the production of the composites are investigated: a coating with binder, a direct reaction and a surface treatment. The advantages as well as the drawbacks of each preparation method are systematically analyzed and addressed. Direct observation, nanoscopic imaging and chemical/elementary analysis are employed to assess the results.

### Anodic oxidation of aluminium surface

**Figure 1:**
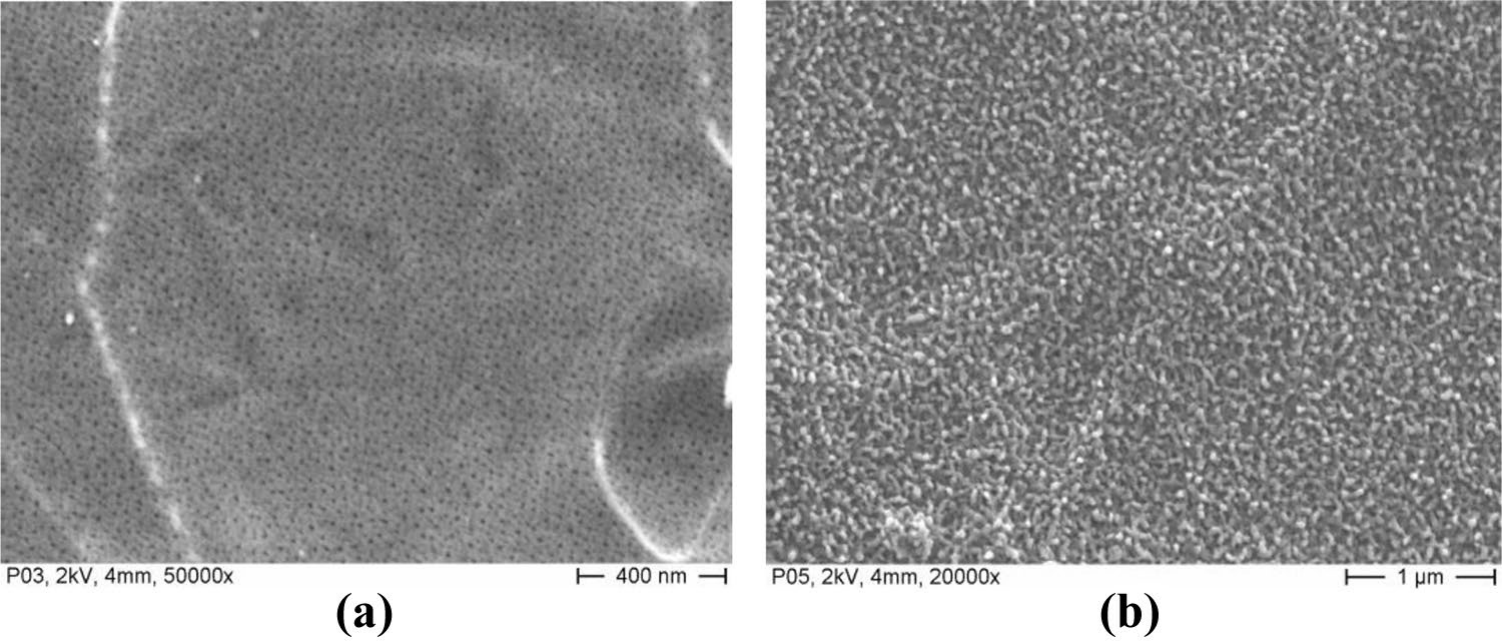
Anodic Oxidising: (**a**) Aluminium oxide Al_2_O_3_, (**b**) Boehmite AlO(OH).

The anodic oxidation as a method of surface conversion treatment was chosen to increase the adherence of the salt hydrates. This surface treatment generates a porous structure of aluminium oxide (alumina) directly on the surface of an aluminium surface. Traditionally this method is composed by two steps: the first step generates a porous aluminium oxide structure while the second creates a coating of aluminium hydroxide that seals the pores. In the following, two approaches are presented to lock the salt without blocking the access to the vapor phase. The first one consists in the use of the honeycombed system of small alumina oxide tubes (Al_2_O_3_) generated during the first step to retain the crystals of adsorbent and increase its adherence to the metal surface. The generated honeycombs show a diameter of about 50 nm and a length of 200 nm (Fig. 1a). As mentioned before, these cavities are usually closed during the second step by a thin layer of boehmite, AlO(OH), sustained by a boiling process of the aluminium oxide tubes. In the second approach, this sealing process is modified so that the salt crystals are captured in an evenly overlaid layer of boehmite (AlO(OH)), which is not intended for sealing in this case. The second step was performed in a saturated solution of the corresponding salt. The described patterns show dimensions in the range of 50–100 nm and appear as spattered drops (Fig. 1b). The surface generated as a result of sealing process, shows an explicit spatial structure with an enhanced contact area. This surface pattern as well as the multitude of their bonding disposition are highly well suited to host and to retain salt crystals. Both described structures appear indeed porous and show small cavities, that seem to be well suited to allow the retention of salt hydrate and the adsorption of vapour by the salt during the operation of the adsorber. However, the EDX element analysis of these surfaces could detect traces of magnesium and sulphur on the surface of boehmite whereas it could not be perceived in case of the alumina surface.

**Figure 2:**
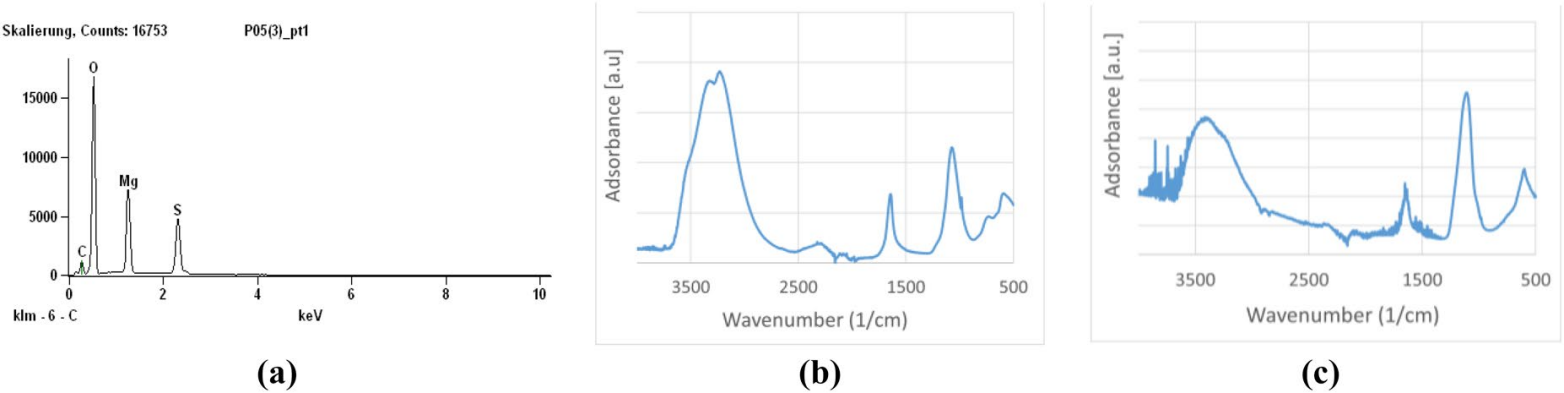
(**a**) EDX of Boehmite covered Aluminium plate, charged with MgSO_4_, (**b**) ATR-FTIR of Boehmite and MgSO_4_ coating, (**c**) ATR-FTIR spectrum of pure MgSO_4_.

ATR-FTIR of the sample confirmed that the element is found as magnesium sulphate (see Fig. 2b). In the spectrum, the peaks at 610–680 and 1080–1130 cm^−1^ characteristic of the sulphate ion with the peaks at 1600–1700 cm^−1^ and 3200–3800 cm^−1^ characteristic of lattice water are visible (see Fig. 2a,c). The presence of the magnesium ion barely modifies the spectrum^54^.

**Figure 3:**
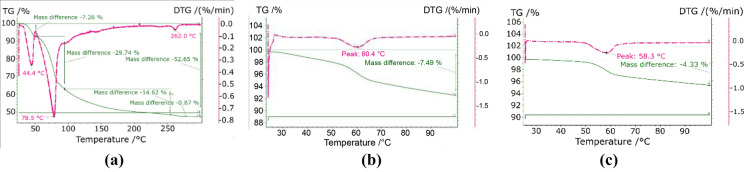
Comparison of the TGA analysis of Boehmite and MgSO_4_: TGA analysis of pure MgSO_4_ (**a**), mixture (**b**) and after rehydration (**c**).

The maintenance of the adsorption properties was verified by TGA. Figure 3b shows a desorption peak at ca. 60 °C. This peak does not correspond to the temperatures of the two peaks observed in the TGA of the pure salt (Fig. 3a). The repeatability of the adsorption–desorption cycle was evaluated, observing the same curve after subjecting the sample to a humid atmosphere (Fig. 3c). The difference observed during the second desorption step may be a result of the dehydration under a flowing atmosphere, because this frequently lead to an incomplete dehydration. The values correspond to roughly 17.9 g/m^2^ during the first dehydration and 10.3 g/m^2^ in the second dehydration.

**Figure 4:**
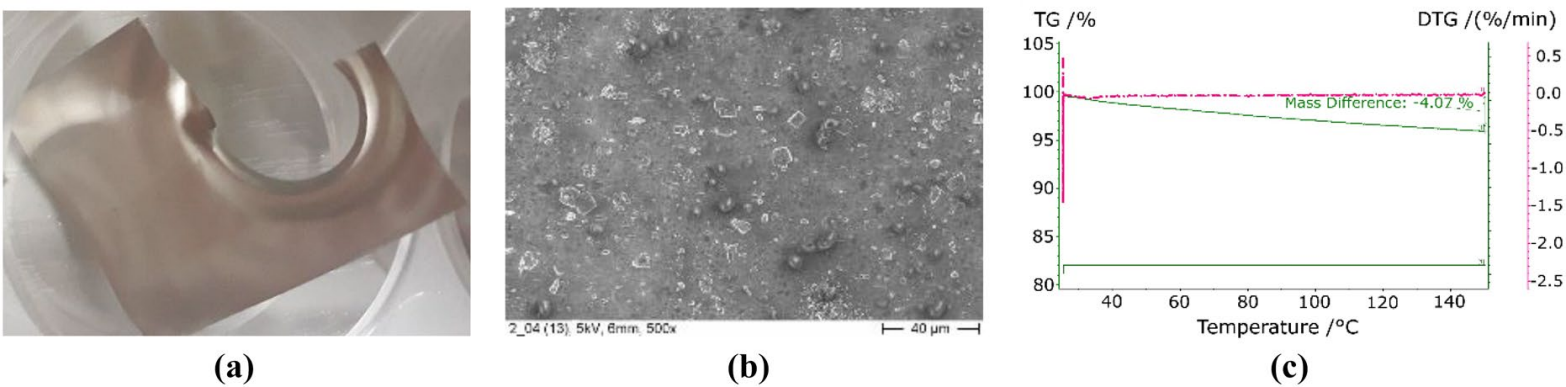
Eloxal with CaCl_2_: real view (**a**), SEM (**b**), and TGA (**c**).

The same method was implemented with calcium chloride as adsorbent. The results are summarized in Fig. 4. The visual inspection of the surface presents a slight variation of the metallic glow. The coat is barely noticeable. SEM confirmed the presence of small crystals uniformly distributed on the surface. However, the TGA does not show any dehydration below 150 °C. This may be due to a proportion of salt compared to the total mass of the substrate so small that it cannot be detected by the TGA.

**Figure 5:**
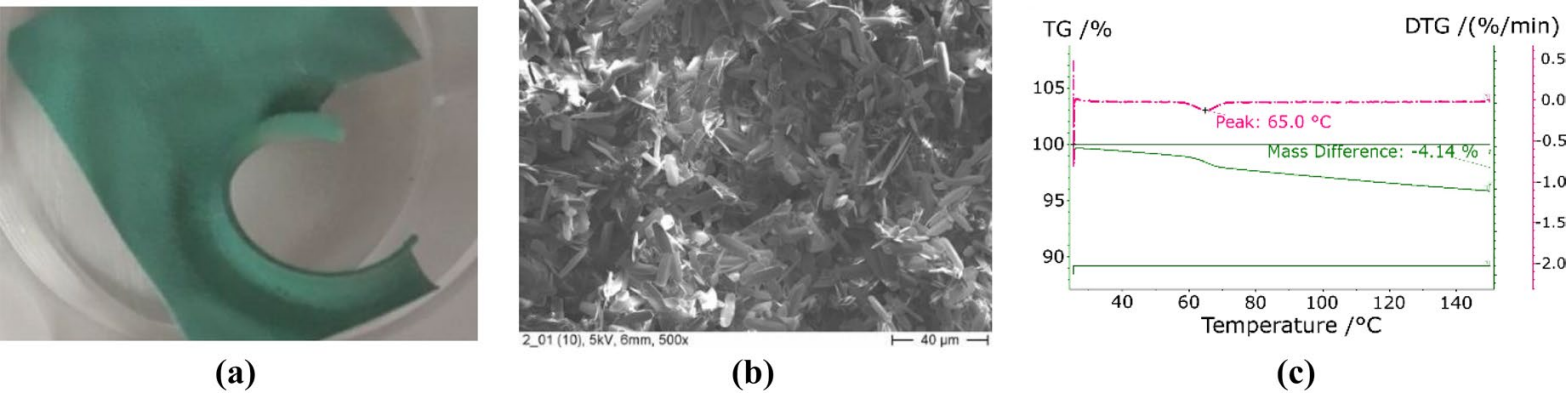
Eloxal with CuSO_4_: real view (**a**), SEM (**b**), and TGA (**c**).

The results of coating with copper sulphate employing anodizing for surface treatment can be found in Fig. 5. In this case, the intended intercalation of CuSO_4_ into the oxide structure of aluminium did not occur. Instead, slackly needles are observed, as they are typically for copper hydroxide, Cu(OH)_2_, which goes along with the typical turquoise dye.

**Figure 6:**
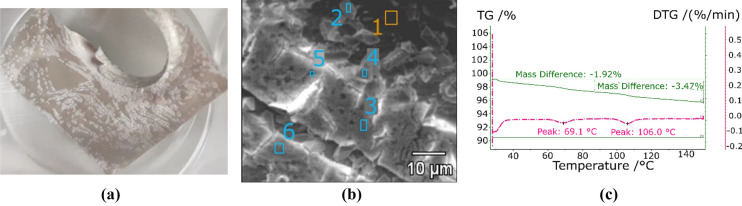
Eloxal with SrCl_2_: real view (**a**), SEM (**b**), and TGA (**c**).

The surface treatment with anodizing was also tested in combination with strontium chloride. The results showed a heterogeneous coating (see Fig. 6a). To determine whether the salt was covering the entire surface an EDX analysis was conducted. The curve for a point in the grey areas (point 1 in Fig. 6b) showed few counts for strontium and many counts for aluminium. This is evidence for a low presence of strontium in the measured area, which is in turn evidence for low coverage with strontium chloride. On the contrary, the white areas returned high counts for strontium and few counts for aluminium (points 2–6 in Fig. 6b). The EDX analysis of the white areas shows darker spots (points 2 and 4 in Fig. 6b) with low counts of chlorine and high counts of sulphur. This could mean the formation of strontium sulphate. The brighter points returned high counts of chlorine and low counts of sulphur (points 3, 5, and 6 in Fig. 6b). It can be interpreted that the main part of the white coating is composed by the expected strontium chloride. The TGA of the sample confirms that interpretation of the analysis, presenting peaks at the characteristic temperatures of pure strontium chloride (Fig. 6c). Their small magnitude can be justified by the small proportion of salt as compared to the mass of the metallic support. The desorbed mass determined in the experiments would correspond to an amount of 7.3 g/m^2^ given per unit of area of adsorber at a temperature of 150 °C.

**Figure 7:**
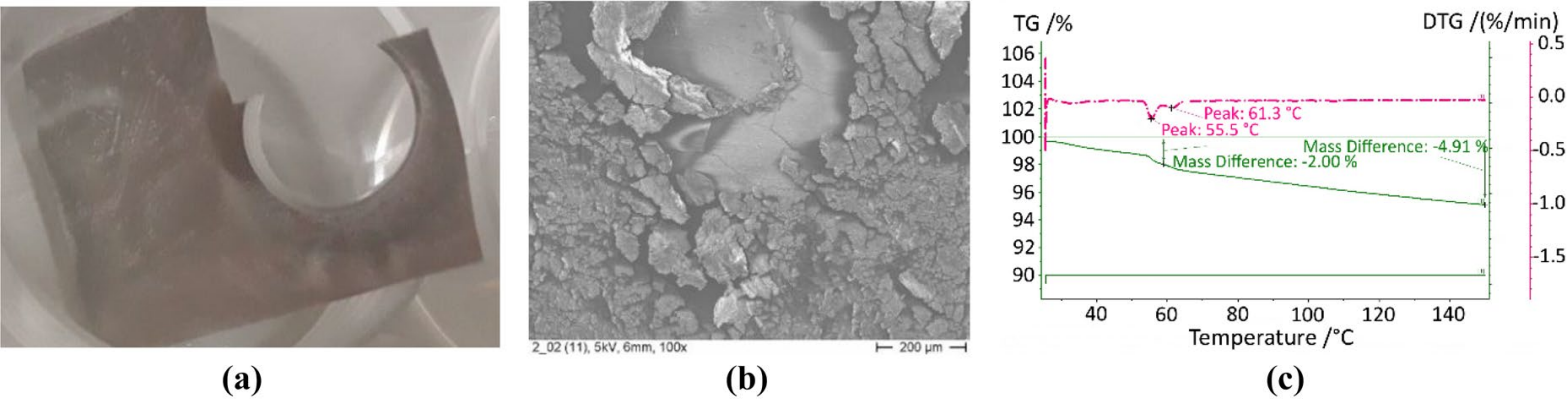
Eloxal with ZnSO_4_: real view (**a**), SEM (**b**), and TGA (**c**).

Coatings of zinc sulphate with an eloxal treatment were also tested. The macroscopic appearance of the coating is that of a very thin and homogeneous layer (Fig. 7a). However, the SEM shows areas of the surface coated by small crystals separated by empty regions (Fig. 7b). The TGA o the coating and substrate was compared to the TGA of the pure salt (Fig. 7c). The pure salt presents a single asymmetric peak at 59.1 °C. The coated treated aluminium shows two small peaks at 55.5 °C and 61.3 °C, indicating the presence of the hydrates of zinc sulphate. The mass difference detected in the experiments correspond to 10.9 g/m^2^ for a dehydration temperature of 150 °C.

### Hydroxyethyl cellulose (HEC)

As in previous applications^53^, hydroxyethyl cellulose was used as binder to increase the adherence and stability of the adsorbent coatings. The material compatibility and impact on the adsorption properties was evaluated by TGA. This analysis was made with reference to the total mass, i.e., the sample included the metallic plate used as substrate for the coating. The adherence was tested with a test based on the cross-cut test defined in the norm ISO2409 (the normed separation between cuts as a function of the thickness and the normed width of the tape could not be satisfied).

**Figure 8:**
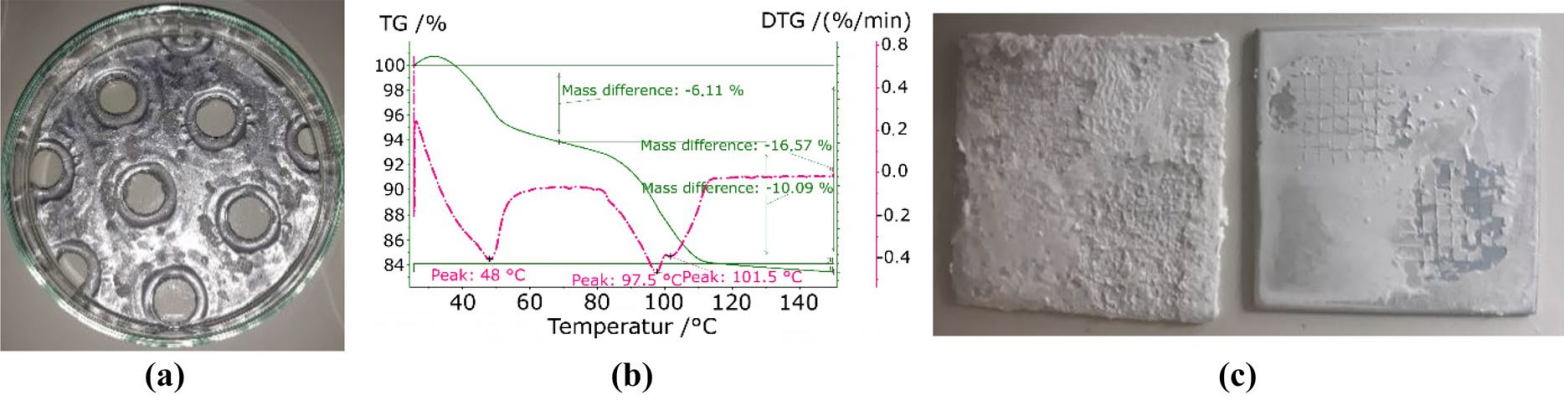
CaCl_2_ coating with HEC: (**a**) fin, (**b**) TGA, (**c**) cross-cut test without (left) and with HEC (right).

The coating of the plates with calcium chloride (CaCl_2_) (see Fig. 8a) led to a heterogeneous distribution, which was not observed in the coating of the pure aluminium used for the cross-cut test. The TGA (Fig. 8b), as compared to the results with pure CaCl_2_, shows a displacement of the two characteristic peaks towards lower temperatures of 40 and 20 °C respectively. The cross-cut test did not allow a fair comparison because the sample with pure CaCl_2_ (sample on the right side in Fig. 8c) was a powder-like deposition, resulting in the removal of the uppermost layer of particles. The results with HEC presented a very thin and uniform coating with a satisfactory adherence. The mass difference represented in Fig. 8b corresponds to 51.3 g/m^2^ per unit area of adsorber at a temperature of 150 °C.

**Figure 9:**
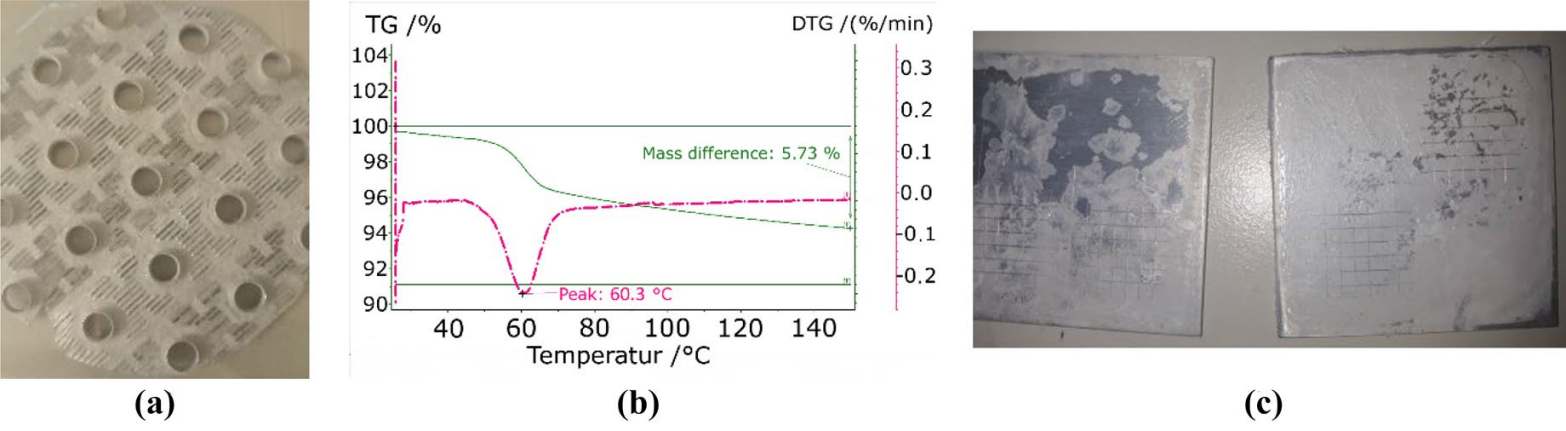
MgSO_4_ coating with HEC: (**a**) fin, (**b**) TGA, (**c**) cross-cut test without (left) and with HEC (right).

Positive results with respect to adherence and uniformity were also obtained, using magnesium sulphate (MgSO_4_) (see Fig. 9). The analysis of the desorption process of the coating shows a single peak at ca. 60 °C. This temperature corresponds to the main desorption step observed during the dehydration of the pure salt, which presents another step at 44 °C. It corresponds to the transition from the hexahydrate to the pentahydrate and is not observed in the case of the coating with binder. The cross-cut test showed an improvement of the distribution and adherence as compared to the coatings obtained with pure salt. The mass difference observed in the TGA-DTC corresponds to 18.4 g/m^2^ given per unit of area of adsorber at a temperature of 150 °C.

**Figure 10:**
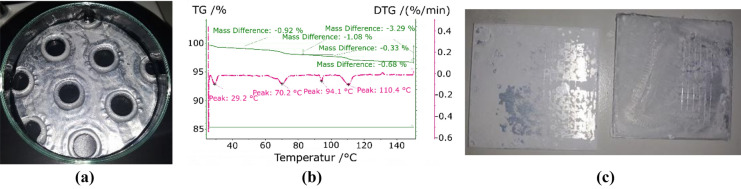
SrCl_2_ coating with HEC: (**a**) fin, (**b**) TGA, (**c**) cross-cut test without (left) and with HEC (right).

Strontium chloride (SrCl_2_) presented a heterogeneous coating over the fin due to the irregularities of its surface (Fig. 10a). However, the results during the cross-cut test resulted in a uniform distribution with highly improved adherence (Fig. 10c). The TGA analysis shows a very small mass difference, surely caused by the low proportion of salt as compared to the metallic support. However, the steps in the curve prove the presence of dehydration processes, although the peaks are deviated with regards to the temperatures obtained in the characterization of the pure salt. The peaks at 110 °C and 70.2 °C observed in Fig. 10b are also detected in the analysis of the pure salt. However, the main dehydration step observed at 50 °C in the pure salt is not reflected in the curves with binder. On the contrary, the mixture with binder presents two peaks at 20.2 °C and 94.1 °C that are not measured with pure salt (Fig. 10b). The observed mass difference corresponds to 7.2 g/m^2^ given per unit of area of adsorber at a temperature of 150 °C.

**Figure 11:**
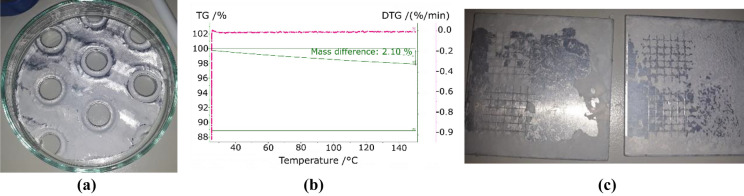
ZnSO_4_ coating with HEC: (**a**) fin, (**b**) TGA, (**c**) cross-cut test without (left) and with HEC (right).

The combination of HEC and zinc sulphate (ZnSO_4_) did not provide acceptable results (Fig. 11). The TGA analysis of the coated metal did not show the presence of any dehydration process. Even though there was an improvement in the distribution and adherence of the coating, its characteristics were still far from optimal.

### Metal fibres

**Figure 12:**
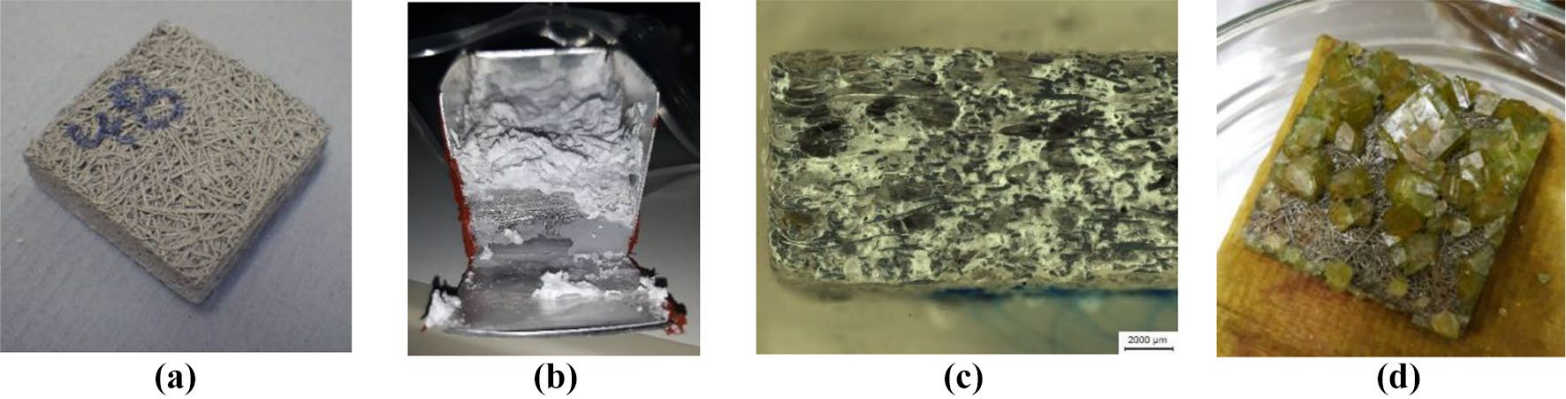
Aluminium fibres coated with (**a**) Al_2_(SO_4_)_3_, (**b**) and (**c**) MgSO_4_ and (**d**) SS-316L with FeCl_2_.

The simplest method to coat metallic fibres with a thin and uniform layer is by wet impregnation (Fig. 12a), which consists in the preparation of the target salt in water solution and the impregnation in the metallic fibres.

Two main challenges were faced in the preparation by wet impregnation. On one side, the surface tension of the salt solution impeded a proper introduction of the liquid in the porous structure. Crystallization on the external face (Fig. 12d) and bubbles trapped inside the structure (Fig. 12c) were only reduced by reducing the surface tension pre-wetting the sample with distilled water. Forcing the dissolution into the sample, either by evacuating the air inside or by creating a current of solution through the structure, are other effective methods to ensure a complete filling of the structure^55^.

**Figure 13:**
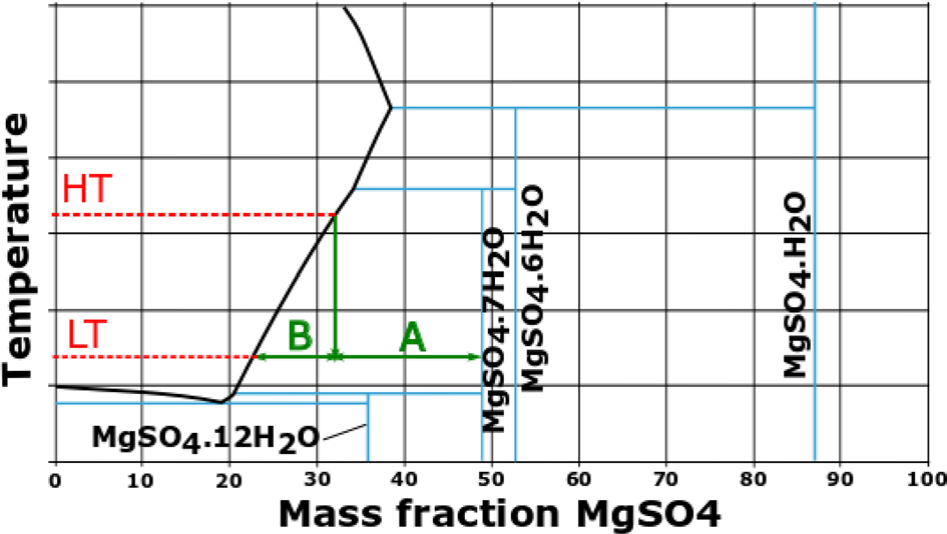
Schematic representation of the crystallization method by cooling and separation of solid and liquid phases with MgSO_4_.

The second challenge faced during the preparation was “skinning” suffered by some of the salts (see Fig. 12b). This phenomenon is characterized by the formation of a dry coat on the surface of the dissolution, which stops the convection-driven drying and starts a diffusion driven process. This second mechanism is much slower than the former one. The consequence is that reasonable drying durations require high temperatures, which increase the risk of formation of bubbles inside the sample. This problem was solved by implementing another crystallization method not based on a change of the concentration (evaporation) but on a change of the temperature (exemplified in Fig. 13 for MgSO_4_).

With this method, a saturated solution of the salt was prepared either at room temperature or at higher temperature (HT). In the first case, crystallization was forced by lowering the temperature below room conditions. In the second case, crystallization happened by letting the sample cool down at room temperature (LT). The result was a mixture of crystals (B) and dissolution (A), whose liquid part was removed with compressed air. This method not only avoided skinning in those hydrates that suffered it, but also decreased the time required for the preparation of other composites. However, removing the liquid with compressed air, contributed to the crystallization of additional salt, which derived into the formation of thicker coatings.

**Figure 14:**
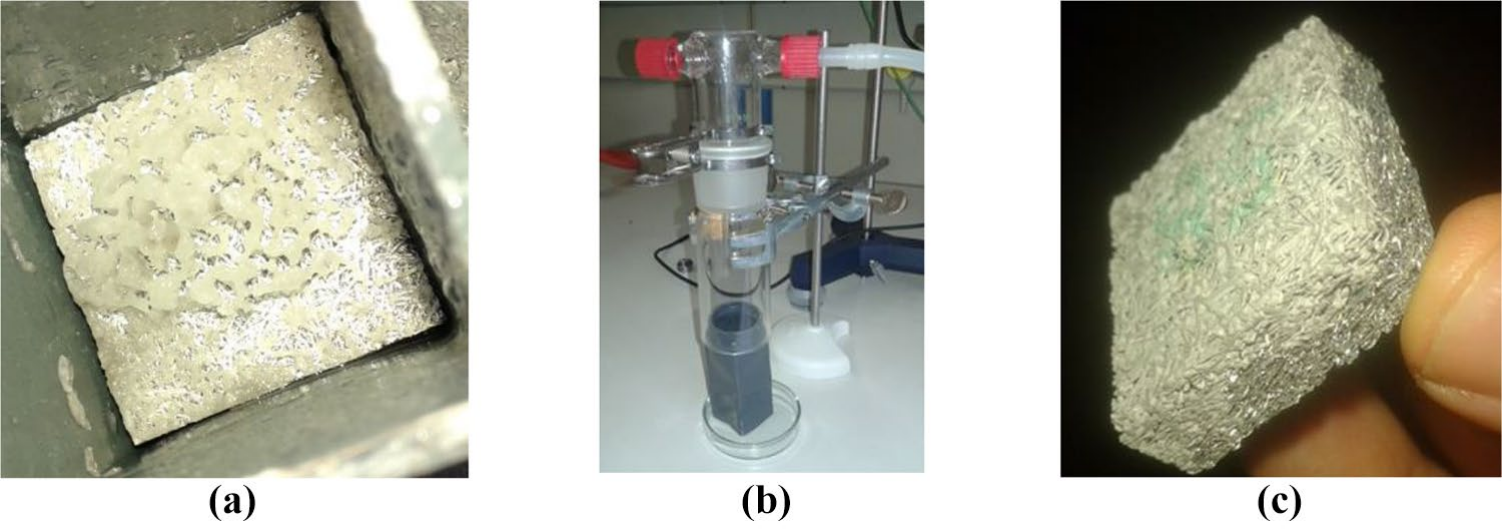
Al_2_(SO_4_)_3_ from acid solution (**a**), synthesis installation (**b**), and result from vapour reaction (**c**).

An alternative method that can be used to coat metallic surfaces consists in the generation of the target salt directly by chemical reaction. Coated heat exchangers produced by the reaction of an acid on the metallic surface of the fins and tubes had several advantages as reported in our previous study^28^. The transference of that method to the fibers resulted in very poor results due to the gases produced in the reaction. The pressure of the hydrogen bubbles built up inside the probe and displaced the products in their way out (Fig. 14a).

A variation of the coating by chemical reaction was implemented to achieve better control of the layer in terms of thickness and distribution. The method consisted in forcing a current of acid mist though the sample (Fig. 14b). This was expected to create uniform coating by reaction with the metal of the substrate. Results were satisfactory but the process was too slow to constitute a valid approach (Fig. 14c). Shorter reaction times could be achieved by local heating.

In order to overcome the drawbacks of the methods presented above, a coating method based on the use of a binder was investigated. HEC was chosen based on the results presented in the previous section. All the samples were prepared with a proportion of 3% wt. binder mixed with the salt. The fibers were pretreated following the same procedure used with the fins, i.e., 15 min immersion in 50% vol. sulfuric acid, followed by 20 s in sodium hydroxide, washed with distilled water, and finally bathed in distilled water for 30 min. In this case, an additional step was added before the impregnation. The samples were shortly submerged in a diluted solution of the target salt and dried at around 60 °C. This process was intended to modify the surface of the metal creating seeding points that would improve the distribution of the coating in the final step. The fiber structures present a side, where the filaments are finer and closely packed and an opposite side, where the filaments are thicker and coarsely distributed. This is a consequence of the fabrication process^52^.

The results with calcium chloride (CaCl_2_) are summarized and illustrated in the images in Table 1. There is a good coverage after the seeding process. Even those filaments that did not have visible crystals on the surface, had reduced their metallic reflection, evidencing a change on the surface finishing. However, after impregnating the sample with the aqueous mixture of CaCl_2_ and HEC and drying at around 60 °C, the coating was concentrated at crossing points of the structure. This is an effect caused by surface tension of the solution. After the immersion, the liquid is retained inside the sample because of its surface tension. This takes place mainly at crossing points of the structures. The finest side of the sample had some of its pores blocked by the salt. There is an increment of mass of 0.06 g/cm^3^ after the coating.

**Table 1:** CaCl_2_ seeding and coating on aluminium fibre structures.

	Seeding	Coating
Fine side		
Coarse side		

Coating with magnesium sulfate (MgSO_4_) yielded higher amounts of salt per unit volume (Table 2). In this case an increment of 0.09 g/cm^3^ was measured. Already the seeding process resulted in the coverage of big areas of the sample. After the coating process salt blocked big areas of the fine side of the sample. Also, some areas of the coarse side are blocked but certain porosity is retained. The formation of salt on crossing points of the structure is easily observed in this case, confirming that the coating process is mainly driven by the surface tension of the liquid and not by an interaction between the salt and the metal substrate.

**Table 2:** MgSO_4_ seeding and coating on aluminium fibre structures.

	Seeding	Coating
Fine side		
Coarse side		

The results with the combination of strontium chloride (SrCl_2_) and HEC presented similar characteristics as the previous examples (Table 3). In this case the finer side of the sample was almost completely blocked. Only a few pores could be seen, which were created during the drying process as the vapor flowed out of the sample. The pattern observed in the coarse side is very similar to that obtained in previous cases, with areas blocked by salt, and fibers completely uncoated.

**Table 3:** SrCl2 seeding and coating on aluminium fibre structures.

	Seeding	Coating
Fine side		
Coarse side		

### Thermal conductivity of coated metal fibres

**Figure 15:**
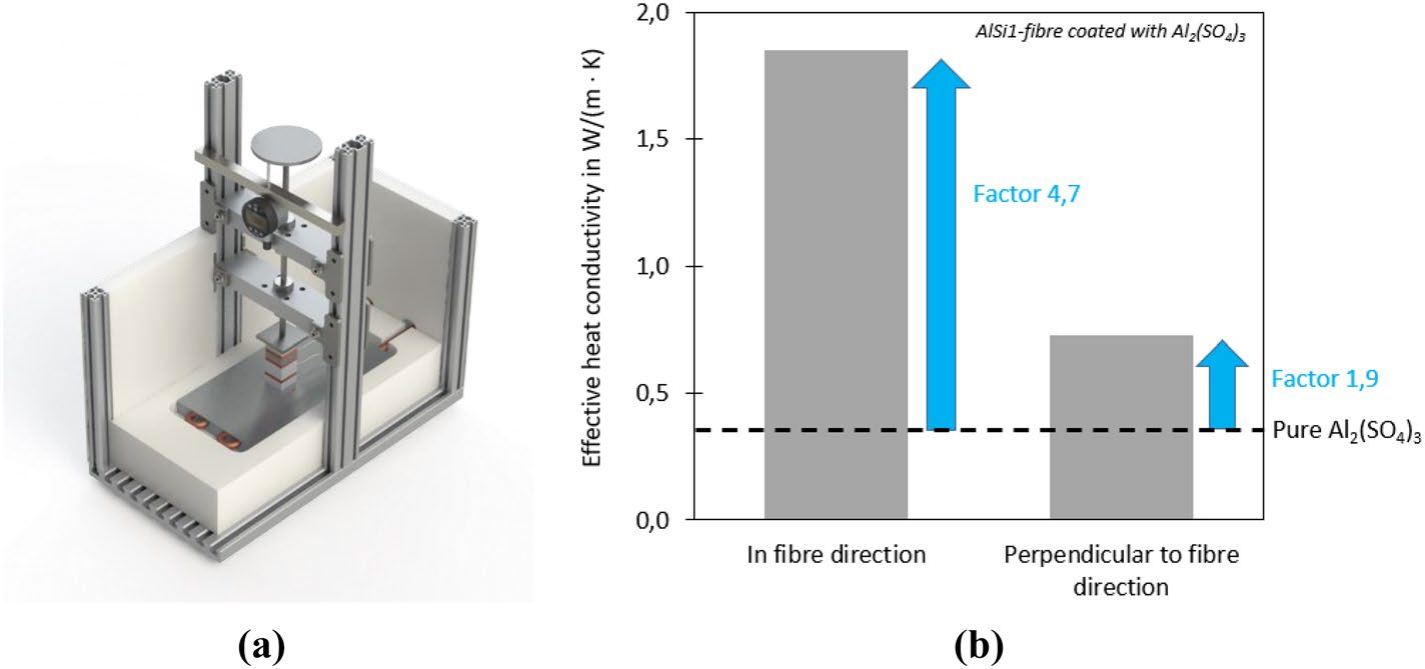
Determination of the effective heat conductivity of aluminium fibre structure coated with Al_2_(SO_4_)_3_. (**a**) experimental setup plate apparatus and (**b**) measurement results compared to pure Al_2_(SO_4_)_3_.

To evaluate the positive effect of the fibre structure regarding the thermal performance of a heat exchanger, the effective thermal conductivity of the coated fibre structure was determined and compared to the pure coating material. The thermal conductivity was measured with a plate apparatus, shown in Fig. 15a, according to ASTM D 5470-2017, applying a reference material with known heat conductivity. Compared to other transient measurement methods this principle is beneficial for porous materials as applied in the current study due to measurement in steady-state and an adequate sample size (footprint of 30 × 30 mm^2^, height of ~ 15 mm). Samples have been prepared for the pure coating material (reference) and the coated fibre structures, both for the measurement in fibre direction and perpendicular to the fibre direction, to assess the influence of the anisotropic heat conductivity properties. The samples were grinded (grain P320) at the surface to minimise the influence of the rough surface due to sample preparation, which is not representative for the structure inside the sample.

The measurement results are shown for the coating of an AlSi1-fibre structure with Al_2_(SO_4_)_3_ in Fig. 12b. The effective thermal conductivity can be increased by the porous fibre structure to a value up to 4.7 times higher than the intrinsic conductivity of aluminium sulfate. As expected, the increase with a heat flux along the fibre direction shows a clearly larger increase. Yet, with a non-optimal fibre orientation the increase is nearly factor 2. Additionally, it has to be taken into account that the reference sample is a non-porous sample without cavities inside the sample, whereas the coated structures already include the cavities to enable a media flow in the structure. The initial fibre structure has a porosity of 81.1% (sample in fibre direction) and 77.4% (sample perpendicular to fibre direction). With cavities (e. g. a loose bulk as applied for zeolithes) the effective heat conductivity of the reference sample would be even lower, which would increase the factor for the heat conductivity improvement. This verifies the improvement of the effective thermal conductivity by the fibre structure. The detailed influence of different parameters e. g. coating thickness, coating distribution of the fibres has to be evaluated in future research.

## Summary and conclusion

In this paper, an approach to overcome the low thermal conductivity of salt hydrates for their implementation in chemical adsorbers has been investigated. Three methods were investigated for the preparation of highly conductive composites in detail: coating with HEC as binder, coatings by direct reaction, and coating with surface treatment of the aluminium substrate by anodic oxidation.

Among the methods analysed in this work, coating combined with surface treatment by anodization of the surface is the most promising approach. The results show the formation of very thin layers of adsorbent with very strong adherence. The TGA of the coatings show the adsorption capabilities of this method showing a negative impact on the formation of hydrates only in the case of calcium chloride. An optimization of the method is required to increase the percentage of mass difference of the composite. From our perspective, the improvement of the coating should begin with an increment of the porosity resulting from the anodizing process. Previous results show that the conditions and additives of the reaction can be modified to duplicate the size of the pores and reach porosities up to 65%^56^.

Coating with HEC as binder presents improved adherence as compared to coatings with pure hydrate. This method can be potentially used with any compound, although the binder can block the adsorption properties of the active material as observed in the case of zinc sulphate. This coating approach constitutes an improvement as compared to the previous encapsulation found in the literature^18,19^, because the direct contact with the surface of the heat exchanger increases the overall heat conductivity, improving the specific power of the adsorber. The transfer of this technique to metallic fibres by immersion and subsequent drying by heating resulted in heterogeneous distributions of the coating due to the influence of the surface tension of the solution. The results presented in this work constitute a broadening of the range of species that can be employed in coated metallic porous structures, as the current state of the art was limited to zeolites or particle beds. This increases the selection of species that can be used in adsorption processes improving the range of temperatures of application. Coatings by wet impregnation or direct reaction in fibres proved to be difficult to control. On the other hand, acid reaction with gases possesses the potential of higher control of the thickness and distribution of the coating. Parameters such as temperature, flow rate, and concentration can be finely tuned to produce optimal results. In future steps, local heating will be supplied to accelerate the reaction and achieve local control of the thickness.

The coating process applied to the metal fibres, either with our without binder, could be improved by a modification of the impregnation and crystallization processes. The crystallization process should happen inside the solution to avoid the influence of the surface tension of the liquid. The problem with that approach is, that the crystallization process is driven by the concentration of the solution (the coat starts forming when the solution saturates), which frequently lead to very thick coats. In order to allow the coating process to yield thinner coats, the concentration of the solution, at which it saturates, should be decreased by the use of lower temperatures. In that case the evaporation of the solvent has to be forced by the reduction of the pressure. The characterization of the thermal conductivity of the coated fibres shows a major increment as compared to the raw adsorbent. This feature, in combination with the characteristic porosity of these structures, evidences the capacity of metal fibres to increase the kinetics and efficiency of adsorbers.

## Data Availability

The datasets generated during and/or analysed during the current study are available from the corresponding author on reasonable request.
